# Diphtheria Outbreak among Persons Experiencing Homelessness, 2023, Linked to 2022 Diphtheria Outbreak, Frankfurt am Main, Germany

**DOI:** 10.3201/eid3103.241217

**Published:** 2025-03

**Authors:** Jonas Haller, Anja Berger, Alexandra Dangel, Katja Bengs, Imke Friedrichs, Christian Kleine, Dorothee Schmidt, Maria Goetzens, Udo Goetsch, Michael Hogardt, Andreas Sing

**Affiliations:** Robert Koch Institute, Berlin, Germany (J. Haller); European Centre for Disease Prevention and Control, Stockholm, Sweden (J. Haller); Gesundheitsamt Frankfurt am Main, Frankfurt am Main, Germany (J. Haller, C. Kleine, D. Schmidt, M. Goetzens, U. Goetsch); Bavarian Health and Food Safety Authority, Oberschleißheim, Germany (A. Berger, A. Dangel, K. Bengs, A. Sing); Laborarztpraxis Rhein-Main MVZ GbR, Frankfurt am Main (I. Friedrichs); Elisabethen Straßenambulanz, Frankfurt am Main (M. Goetzens); University Hospital Frankfurt, Frankfurt am Main (M. Hogardt)

**Keywords:** Corynebacterium diphtheriae, diphtheria, molecular epidemiology, outbreak, multilocus sequence typing, persons experiencing homelessness, migrants, public health, bacteria, Frankfurt am Main, Germany

## Abstract

After 3 cases of *Corynebacterium diphtheriae* infection associated with intravenous drug use among persons experiencing homelessness (PEH) were reported to the Health Protection Authority in Frankfurt am Main, Germany, in 2023, we examined pathogen spread among PEH. Furthermore, we investigated a possible link with the 2022 outbreak of diphtheria in Europe. From swab samples collected during August–November 2023 from 36 PEH and cutaneous lesions, we detected 3 additional cases of cutaneous toxigenic *C. diphtheriae*. Sequence type 574 was identified in 5 case-isolates and is genetically associated with 1 of the predominant clusters in identified in the 2022 outbreak. Our findings demonstrate the need for increased detection and monitoring of cutaneous diphtheria and boosting immunity against diphtheria in groups with increased risk for infection. Genomic analyses are valuable for identifying genetic relationships between outbreaks, even when epidemiologic data are scarce.

Diphtheria is a vaccine-preventable disease, caused most often by toxigenic strains of *Corynebacterium diphtheriae*. Diphtheria is a worldwide public health threat that may affect the respiratory tract, especially the larynx or the skin, and may rarely cause ocular, otic, or genital disease (*1*–*3*). *C. diphtheriae* is almost exclusively harbored by humans, and transmission is primarily through airborne respiratory droplets or direct contact with cutaneous lesions or contaminated objects and fomites (*1*,*3*). The incubation period for diphtheria is typically 2–5 days (range 1–10 days) (*3**–**5*). In wound infections, *C. diphtheriae* is frequently found along with other skin pathogens such as *Streptococcus pyogenes* or *Staphylococcus aureus* (*6*).

Cutaneous wounds colonized or infected by toxigenic *C. diphtheriae* are a concerning potential source of severe respiratory diphtheria infections, which can lead to high mortality rates when untreated (*7*). The primary treatment available to neutralize the effects of the toxin is equine diphtheria antitoxin, which should ideally be administered within 48 hours of initial symptom onset. However, the production, supply, and availability of equine diphtheria antitoxin have declined over the past decade because of low demand across Europe, leading to shortages in many countries in Europe (*4*,*8*,*9*).

The World Health Organization aimed to eliminate diphtheria by 2000; thus, a worldwide immunization program was initiated in the late 1970s. As a result, diphtheria cases have substantially decreased in many countries (*10*,*11*). Full vaccination, typically requiring >3 doses of a diphtheria toxoid–containing vaccine, provides robust protection: 87% against symptomatic disease and 93% against death (*5*).

In Europe, diphtheria affects mainly persons insufficiently immunized before travel to regions where diphtheria is endemic (e.g., Africa, the Eastern Mediterranean, and Southeast Asia) and vaccination coverage is historically low (*1*,*4*,*12*–*15*). In 2015, the European Centre for Disease Prevention and Control issued a rapid risk assessment concerning the potential occurrence of cutaneous diphtheria among migrants originating from diphtheria-endemic regions and unvaccinated travelers returning from those areas (*16*). Since 2022, cases of cutaneous diphtheria have notably increased across numerous countries in Europe among migrant populations, particularly affecting young men originating from Syria and Afghanistan. Furthermore, instances of respiratory diphtheria, some resulting in fatalities, have also been documented (*1*–*24*).

Outbreaks have, however, also been observed in other population groups. An outbreak of cutaneous diphtheria caused by toxigenic *C. diphtheriae* in a group of persons with alcohol use disorder in Sweden has been described, and nontoxigenic *C. diphtheriae* has frequently been found in the wounds of persons associated with intravenous drug use (IVDU) (*19*). Because persons with alcohol and substance use disorders often have difficulty accessing reliable medical care, underreporting might be a substantial problem, potentially increasing the risk for diphtheria transmission (*18*–*21*).

In Germany, diphtheria is a notifiable disease. In February 2023, a case of respiratory diphtheria was reported to the Health Protection Authority in Frankfurt am Main (hereafter abbreviated as Frankfurt), followed by 2 cases of cutaneous diphtheria in June and July 2023. In both cases, the infections were characterized by superficial ulceration and abscess formation, primarily in the groin area. Both patients had mixed wound infections with *S. aureus*, *S. pyogenes*, or both. One patient died of *S. aureus* sepsis. For all 3 patients, the time since last vaccination against *C. diphtheriae* was either unknown or confirmed to be >10 years. The patients experienced homelessness, spent time around the central station in Frankfurt, and engaged in IVDU. None of the 3 patients had traveled outside of Germany within 10 days before the sample collection date. Unfortunately, further epidemiologic investigation and contact tracing was not possible.

Until the report of a respiratory diphtheria case in a person with IVDU and experiencing homelessness in February 2023, no isolates from non–travel-related patients with *tox* gene have been reported in Frankfurt since 2014. The PCR test for the diphtheria *tox* gene in all 3 isolates was positive; the number of cases in the nonmigrant population of Frankfurt was higher than expected. We investigated the extent of the outbreak and its genetic relationship with other cases reported in Germany.

## Material and Methods

### Case Definition

We defined a suspected case as illness in any person experiencing homelessness who had typical skin lesions and was notified during August 1, 2023–October 31, 2023, while residing in Frankfurt am Main and without having returned from a diphtheria-endemic area within the 10 days before testing. We defined a probable case as illness in a person with a suspected case and a positive culture result for *C. diphtheriae*. We defined a confirmed case as illness in any person with a probable case and a *C. diphtheriae* *tox* gene–positive (*tox*+) isolate, which includes the 3 confirmed case-patients reported earlier in the year (February, June, and July 2023). We did not classify probable cases with a *C. diphtheriae* isolate that tested negative for the *tox* gene (*tox*–) as confirmed cases. Although the definitions of probable and confirmed case were applied to the August–October 2023 period, the inclusion of earlier cases enabled comprehensive knowledge of the outbreak.

### Notifiable Disease

The first 3 PCR-positive (*tox*+) cases during February–July 2023 were reported to the Health Protection Authority in Frankfurt by various local hospitals. The first case was identified by PCR and clinical signs/symptoms but without any further typing. The other isolates were sent to the German National Consiliary Laboratory for Diphtheria at the Bavarian Health and Food Safety Authority and World Health Organization Collaborating Centre for Diphtheria (Landesamt für Gesundheit und Lebensmittelsicherheit) in Bavaria for toxigenicity testing and whole-genome sequencing (WGS).

Patients were isolated and treated in single rooms in the hospitals and, when possible, interviewed by staff of the Health Protection Authority with regard to possible contact persons. We collected information on age, sex, duration of illness at first visit, clinical manifestations, and vaccination history from medical records.

### Epidemiologic Investigation

For further investigation and to determine the extent of the outbreak, we initiated active case finding in collaboration with 3 aid organizations for persons experiencing homelessness (PEH), persons with and without concomitant drug dependence close to the central railway station, or both, as follows. First, we gave the aid organizations information about the potential risk of PEH contracting diphtheria. Then, during August 2023–October 2023, the public health authority of Frankfurt supplied the homeless aid organizations with the necessary material to systematically screen all patients with skin lesions for *C. diphtheriae*. Patients with open skin wounds were medically examined by a doctor and informed about the screening, which was followed by simultaneous collection of wound and throat swab samples. Last, persons exhibiting clinical signs of respiratory diphtheria or severe skin lesions were promptly directed to the nearest hospital for further evaluation to confirm or rule out suspicion of diphtheria and to receive treatment. In addition to the age, sex, and location of the affected persons, we also received information about their medical history, including possible dependency disorders, vaccination status, and signs/symptoms.

### Vaccination

Among the ≈300 PEH in Frankfurt, vaccination coverage against diphtheria is mostly unknown because of lost vaccination cards and sporadic visits to medical aid facilities. Therefore, we conducted a vaccination campaign in collaboration with the Elisabethen Street Ambulance (ESA), one of the aid organizations for PEH. In December 2023, the local health authorities provided ESA with 50 doses each of diphtheria-tetanus and influenza vaccine. To ensure comprehensive coverage, a mobile team of streetworkers and a medical doctor from ESA made weekly outreach visits to the central railway station area via a specialized bus to reach persons who did not have easy access to the ESA facility

Furthermore, we conveyed essential knowledge about diphtheria to the staff members within various homeless aid organizations, raising awareness about diphtheria and encouraging them to confirm their vaccination status. Following the Germany national guidelines for a proven case of diphtheria, we recommended that in-contact persons whose last vaccination for diphtheria was >5 years ago should receive a booster, as opposed to the standard interval of 10 years for boosters (*25*). Staff members of the aid organizations were instructed to self-report any typical signs/symptoms to their family doctor for further diagnosis after having close contact with persons with suspected cases.

### Microbiological and Genomic Analyses

At the Laborarztpraxis Rhein-Main MVZ GbR in Frankfurt, we cultured samples on 5% sheep blood and serum tellurite agar plates (both BD, https://www.bd.com). After colony incubation for a minimum of 3 days, we subjected colonies that exhibited characteristics suggestive of coryneform bacteria to matrix-assisted laser desorption/ionization time-of-flight mass spectrometry with VITEK MS PRIME (bioMérieux, https://www.biomerieux.com), followed by microbiological susceptibility testing, which was performed according to the guidelines from the European Committee on Antimicrobial Susceptibility Testing for all isolates (https://www.eucast.org/clinical_breakpoints) (*1*,*17*,*26*).

To verify toxigenicity and perform WGS, we sent all cultivated *C. diphtheriae* isolates except the respiratory *C. diphtheriae* isolate of the first observed case to the German National Consiliary Laboratory on Diphtheria at the Bavarian Health and Food Safety Authority and World Health Organization Collaborating Centre for Diphtheria in Bavaria. The German National Consiliary Laboratory on Diphtheria verified toxigenicity by using real-time PCR (*27*) and the optimized Elek test (*28*), after which WGS was performed on 5 *tox*+ *C. diphtheriae* strains (*16*). We analyzed WGS data by using multilocus sequence typing (MLST) of target loci *atpA*, *dnaE*, *dnaK*, *fusA*, *leuA*, and *odhA* (*25*) and also by core-genome MLST (cgMLST) by using the *C. diphtheriae* cgMLST scheme of 1,553 target loci (*17*) implemented in Ridom SeqSphere+ software (Ridom GmbH, https://www.ridom.de) for centralized complex type (CT) nomenclature and minimum spanning tree visualization. We performed microbiological susceptibility testing according to the guidelines from the European Committee on Antimicrobial Susceptibility Testing for all isolates (https://www.eucast.org/clinical_breakpoints) (*29*).

We defined a genetic cluster/relationship as cgMLST profiles of isolates with a common CT, with the empirical threshold of a maximum of 14 allelic distances of the total 1,553 typed loci to the CT-founder isolate that established the CT. We included published *C. diphtheriae* isolates of the same sequence type (ST) from Frankfurt and Europe in the cgMLST analysis for genetic relatedness.

## Results

### Outbreak Outline

**Figure 1:**
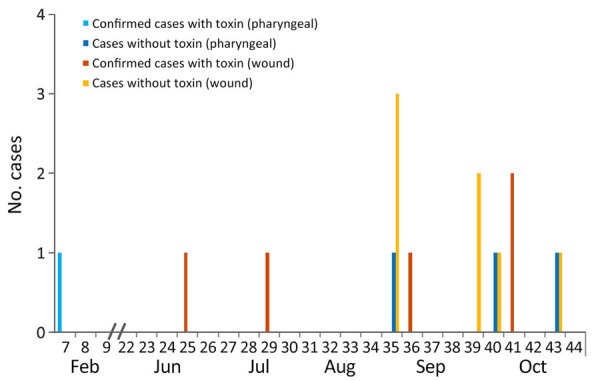
Details on 16 cases of dipththeria in persons experiencing homelessness, by sampling week, toxigenicity (positive/negative), and place of infection, Frankfurt am Main, Germany, February–October 2023.

During August 1, 2023–October 31, 2023, a total of 36 suspected cases were identified. None of the patients refused testing. Of the 36 suspected cases, 13 had probable cases, determined by a positive culture for *C. diphtheriae*. Three confirmed cutaneous diphtheria cases were identified, along with the 3 previously reported cases from earlier in the year, bringing the total number of confirmed cases to 6: 1 respiratory case and 5 cutaneous cases. Of the 10 nonconfirmed case-patients harboring *tox*– *C. diphtheriae* isolates, 7 swab samples were from wounds (Figure 1). Except for the first reported case in February 2023, no patient exhibited clinical signs of respiratory diphtheria. Only 1 patient exhibited severe skin inflammation and was hospitalized (Figure 1). Other skin pathogens (e.g., *S. pyogenes* or *S.* aureus) were isolated from most swab samples. All strains were resistant to trimethoprim/sulfamethoxazole, but we did not observe any antimicrobial resistance against benzylpenicillin (high dosage), erythromycin¸ clindamycin, or tetracycline.

We describe all 6 confirmed cases of diphtheria detected in 2023 because they belonged to 1 outbreak. Most confirmed (5/6) and probable (12/16) case-patients were male; median age was 40 years for the probable case-patients (interquartile range 38–43) (Table). Country of origin was Germany (n = 8), Poland (n = 4), or unknown (n = 4). All 16 patients with a positive culture for *C. diphtheriae* experienced homelessness. Among them, 10 were associated with IVDU, 6 had an alcohol dependency disorder, and 4 had both dependency disorders. The exact date of symptom onset could not be determined because most wound infections had occurred weeks to months earlier. Vaccination status was also unknown because of missing vaccination records. No deaths were reported, and no secondary cases were detected among persons working in the health aid organizations (Table). Furthermore, no further cases of wound/pharyngeal diphtheria in the general population of Frankfurt were notified during the study period.

**Table:** Demographic and clinical characteristics of 16 *Corynebacterium diphtheriae* cases among persons experiencing homelessness, Frankfurt am Main, Germany, February–October 2023*

Case no.	Demographics				Toxigenic status			Symptoms			Substance abuse	
	Age group, y	Sex	Country of origin		Pharynx	Wound		Respiratory	Wound		Alcohol	IV drugs
1†	26–35	F	Germany		+	UNK		Y	Y		N	Y
2†	26–35	M	Afghanistan		–	+		N	Y		N	Y
3†	36–45	M	Germany		–	+		N	Y		Y	Y
4†	>60	M	Poland		–	+		N	Y		Y	N
5†	36–45	F	Poland		–	+		N	Y		Y	N
6†	46–60	M	Poland		–	+		N	Y		Y	N
7	26–35	M	Germany		–	–		N	Y		N	Y
8	36–45	M	Germany		–	–		N	Y		Y	Y
9	36–45	F	Germany		–	–		Y	Y		Y	N
10	46–60	F	UNK		–	–		N	Y		Y	N
11	36–45	M	UNK		–	–		Y	Y		N	Y
12	46–60	M	UNK		–	–		N	Y		Y	Y
13	46–60	M	Poland		–	–		N	Y		Y	N
14	46–60	M	Germany		–	–		Y	Y		N	Y
15	36–45	M	Germany		–	–		N	Y		Y	Y
16	36–45	M	Germany		–	–		N	Y		N	Y

*Probable and confirmed cases are shown. IV, intravenous; UNK, unknown. †Previously reported confirmed cases.

### Laboratory Analysis and Phylogeny

**Figure 2:**
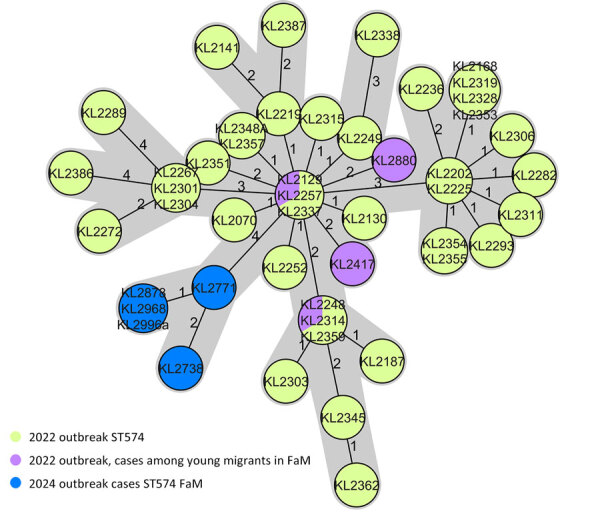
Minimum-spanning tree of core-genome multilocus sequence type analysis of toxigenic *Corynebacterium diphtheria,* with 1,553 target loci of the whole-genome sequencing-obtained genomes of the toxigenic *C. diphtheriae* isolates from an outbreak in 2023 compared with genomes of an outbreak cluster of the same ST (ST574) from a previously reported outbreak among migrants (*23*). Single-linkage allelic distances are illustrated as a measure of genetic related. FaM, Frankfurt am Main; ST, sequence type.

In 5 of the toxigenic outbreak isolates, MLST identified the common ST574. Therefore, those isolates were analyzed by cgMLST and results were compared with the database of sequenced *C. diphtheriae* genomes of the Consiliary Laboratory on Diphtheria. The 5 ST574 isolates showed very close genetic relatedness; total allelic distances from each other were 0–4. Furthermore, the isolates were genetically linked to a cluster of ST574 isolates from the 2022 outbreak among migrants in Europe (*23*) (Figure 2), which also comprised 4 cases among young migrants from the same geographic region of Frankfurt. The 5 current outbreak isolates from PEH in Frankfurt showed a total of 4–13 allelic distances from the migration-associated isolates of that cluster and, within that, 4–8 allelic distances from the Frankfurt-based young migrants (Figure 2). All isolates showed common CT 79, which means that each isolate that harbors the CT has an allelic distance less than or equal to the CT threshold of 14 alleles to the CT-founder isolate that established the CT.

### Treatment and Isolation of Case-Patients

In accordance with the National Guidelines (*25*), case-patients received azithromycin (500 mg/d for 3 days). Azithromycin was preferred to other antimicrobial drugs because its treatment duration is shorter and it needs to be administered only once per day. Because of difficult living conditions, only wound coverage and antimicrobial treatment were provided; no swab samples were collected to confirm pathogen eradication.

### Outbreak Intervention

By the end of January 2024, a total of 26 PEH had received 1 dose of diphtheria-tetanus vaccine and 1 dose of influenza vaccine, administered according to the national recommendations for vaccinations in Germany. We have no records of how many persons out of the study population refused vaccination.

## Discussion

In recent decades, the incidence of toxigenic *C. diphtheriae* cases in Germany has been low because most persons are vaccinated. However, sporadic cases of imported diphtheria in travelers (*30*–*32*) show that booster vaccination is needed to provide sustained protection against the disease. In PEH who have an IVDU or alcohol dependency disorder, single cases and outbreaks have been reported repeatedly (*33*,*34*). Among PEH and persons who use substances, most cases were cutaneous wound infections colonized by nontoxigenic *C. diphtheriae* (*17*,*35*,*36*).

The unexpected notification of 3 cases of *tox*+ diphtheria in PEH in Frankfurt in early 2023 was the reason for initiating active case finding among PEH in Frankfurt. Our study identified predominantly *tox*− strains, with 6 *tox*+ cases among PEH in Frankfurt. That pattern aligns with findings from other studies in which cutaneous manifestations of *C. diphtheriae* rather than pharyngeal infections were found to be prevalent, particularly among at-risk groups (*37*–*39*). Cutaneous diphtheria is often observed in populations with limited healthcare access, including those with high rates of alcohol dependency and IVDU, because wounds can serve as entry points for infection (*37*,*40*). The skin lesions make person-to-person transmission easier and can harbor both toxigenic and nontoxigenic strains, increasing the risk for broader community spread (*37*).

Active case finding, in collaboration with medical aid facilities, proved essential for identifying and managing diphtheria cases among PEH. Those facilities, often the primary contact points for vulnerable populations, provided a trusted environment that enable testing, treatment, and vaccination efforts. Given the limited healthcare access and high prevalence of comorbid conditions such as substance use disorders within this population, conventional healthcare systems alone may struggle to effectively reach such persons (*40*,*41*). By engaging medical aid organizations and outreach services, we were able to reach persons whose illness might otherwise remain undiagnosed, thereby reducing potential transmission risks and improving outbreak containment.

Genetic analysis revealed notable similarity among isolates from PEH, suggesting transmission within this group despite the absence of clearly documented direct contacts. Sequence-based comparisons showed that cases shared close genetic profiles (AD 0–4), indicating the likely circulation of *C. diphtheriae* strains (ST574) within that population. That finding highlights the value of genomic surveillance as an epidemiologic tool, providing insights into transmission pathways that may not be captured by standard contact tracing alone and underscores the value of targeted interventions within at-risk populations to mitigate further spread.

A plausible epidemiologic link between the diphtheria outbreak among migrants in 2022 and the recent outbreak among the community of PEH in Frankfurt is suggested by identification of a ST574 cluster; the close genetic relationship that was subsequently identified between ST574 isolates from the 2023 outbreak and ST574 isolates from the 2022 outbreak among young migrants; and the close relationship of ST574 with CT-79. Our hypothesis is further supported by epidemiologic evidence indicating mobility and intermingling between those populations, particularly within urban centers (*42*). The genetic congruence between the isolate genomes implies a direct or indirect transmission pathway, whereby ST574 was introduced from case-patients from the 2022 outbreak into the community of PEH, either through direct contact or within shared environments, such as shelters, aid facilities, or communal spaces.

During the diphtheria outbreak in Frankfurt, the treatment strategy of azithromycin once daily was preferred over multiple daily doses of erythromycin or penicillin; outcomes were positive (*1*,*43*). Both the national guidelines for Germany (*29*) and the recently published World Health Organization guidelines (February 2024) recommend the primary use of azithromycin to treat confirmed cases (*44*). That regimen, with its shorter duration and once-daily dosing, was specifically chosen to improve treatment compliance.

Improving data on vaccination coverage among PEH is essential. In this study, we were able to vaccinate only 26 of ≈300 persons in our potential target group. Many patients faced challenges with regard to adhering to further treatments (e.g., vaccinations), potentially influenced by underlying mental health conditions and other socio-environmental factors. Understanding barriers to vaccine uptake in this community is crucial. A mixed-methods approach involving qualitative interviews with PEH and outreach staff could identify specific challenges (e.g., limited vaccine access, hesitancy, or inability to track vaccinations). Findings could guide strategies to improve access (e.g., mobile vaccination units) and education to address vaccine concerns. Furthermore, obtaining reliable data on the vaccination status of PEH was difficult because of a lack of vaccination documents and a national vaccination register in Germany. Similar problems were encountered during investigations of the outbreak among migrants in 2022 (*1*,*16*,*23*); migrants receive the basic vaccinations in the initial reception facilities in Germany.

In conclusion, our study underscores the challenges inherent in investigating and controlling diphtheria outbreaks among PEH in a bustling urban center such as Frankfurt am Main. Our findings demonstrate that active case finding for diphtheria among persons in those populations is both feasible and more successful when conducted in collaboration with medical aid facilities dedicated to serving them. By leveraging trusted access points, early detection and intervention efforts can reach at-risk persons more effectively, underscoring the value of engaging community health resources.

Sustained awareness and vigilance are crucial, particularly within medical aid facilities serving high-risk populations. Those facilities, in collaboration with public health organizations, must establish and maintain ongoing partnerships. Such continuous engagement enables both parties to stay informed about emerging public health threats and enhances their preparedness for potential outbreaks. That approach ensures that healthcare providers and the community are more proactive and ready to respond to future infectious disease risk.

Last, molecular typing has proven to be an invaluable asset for providing information about transmission pathways within at-risk populations. By analyzing the genetic profiles of diphtheria strains, health authorities can gain critical insights into potential links between cases, even when direct contact tracing is not possible, thereby enhancing knowledge of disease spread and the effectiveness of outbreak control efforts.

Appendix Additional information for study of diphtheria outbreak among persons experiencing homelessness linked to 2022 diphtheria outbreak in Frankfurt am Main, Germany.
